# Positive estrogen receptor status is a poor prognostic factor in node-negative breast cancer: An observational study in Asian patients

**DOI:** 10.1097/MD.0000000000025000

**Published:** 2021-03-19

**Authors:** Eun Jung Jung, Ju-Yeon Kim, Jae-Myung Kim, Han Shin Lee, Seung-Jin Kwag, Ji-Ho Park, Taejin Park, Sang-Ho Jeong, Chi-Young Jeong, Young-Tae Ju, Young-Joon Lee, Soon-Chang Hong

**Affiliations:** a Department of Surgery, Gyeongsang National University School of Medicine and Gyeongsang National University Changwon Hospital, Changwon; b Department of Surgery, Gyeongsang National University School of Medicine and Gyeongsang National University Hospital, Jinju, Republic of Korea.

**Keywords:** breast neoplasm, estrogen receptor, lymph node, prognosis

## Abstract

This study evaluated the outcomes and prognostic factors for breast cancer according to initial lymph node (LN) status. Among patients with LN-negative breast cancer, we also focused on the prognostic value of estrogen receptor (ER) status.

Medical records were retrospectively reviewed for 715 patients who underwent curative surgery for breast cancer between January 2005 and December 2015 at a single Korean institution. We evaluated factors that were associated with metastasis-free survival (MFS) according to LN status.

Among the 715 patients (age: 28–87 years), 458 patients (64.1%) did not have axillary LN metastasis. Relative to patients without LN metastasis, patients with LN metastasis had larger tumor sizes and higher histological grades. Among patients with no LN metastasis, ER positivity was associated with non-significantly poorer MFS than ER negativity (mean survival: 138.90 months vs. 146.99 months, *p* = .17), and patients with LN-negative ER-positive disease had MFS rates of 91.7% at 5 years and 74.5% at 10 years. Among patients with LN-negative ER-positive disease, a poor prognosis was significantly associated with larger tumor size (≥2 cm, *P* = .03) and older age (≥50 years, *P* = .03).

These results indicate that the risk of metastasis increases over time for patients with LN-negative ER-positive breast cancer, and especially for older patients or patients with larger tumors.

## 1 Introduction

Breast cancer is the most common type of cancer and the main cause of cancer-related deaths among women, with an estimated 2,400,000 incident cases in 2015.^[1]^ In 2015, approximately 90% of newly diagnosed breast cancers in Korea were stage I–II disease, which is primarily related to early diagnosis and improvements in treatment. The 5-year relative survival rate is approximately 98.4% for women with localized breast cancer, although the rates decrease to approximately 90.7% for patients with regional involvement and 39.3% for patients with distant metastasis.^[2]^

The presence or absence of axillary lymph node (LN) metastasis is the most potent prognostic factor for primary breast cancer patients, and the clinical outcomes are generally associated with the number of metastatic LNs. Furthermore, the 5-year relapse free survival rate is approximately 80% among node-negative patients, which indicates that 20% of patients in this low-risk group still experience relapse.^[3]^ Thus, it would be useful to identify factors that predict primary tumor growth and/or metastasis, which would help identify node-negative patients who could benefit from more aggressive therapy. However, few studies have specifically evaluated these factors in node-negative patients.^[4–6]^ A recent review of studies with large patient sample sizes and prolonged follow-up periods revealed that survival outcomes were significantly related to tumor size, histological grade, vascular invasion, Ki-67 index, cathepsin-D concentration, S-phase fraction, and mitotic index.^[7]^ However, mixed results were observed for the relationship between survival and estrogen receptor (ER) status, and survival was not associated with human epidermal growth factor receptor 2 (HER-2) status.^[7]^ Therefore, the present study aimed to evaluate the outcomes and prognostic factors according to initial LN status among patients with breast cancer, especially regarding ER status. This information might help clinicians predict disease progression and select appropriate treatments while effectively balancing the risks, costs, and benefits.

## 2 Methods

This retrospective study evaluated Korean women who were diagnosed with primary breast cancer and underwent curative surgery between January 2005 and December 2015 at a single institution. Patients were excluded if they had distant metastasis at the diagnosis, received neoadjuvant chemotherapy, had synchronous bilateral breast cancer, or were followed for <6 months. Based on those criteria, 715 patients were considered eligible. The retrospective study protocol was approved by our institutional review board (GNUH 2018-10-017) and complied with the tenets of the Declaration of Helsinki. The requirement for informed consent was waived based on the retrospective design.

After surgery, the patients were recommended to undergo adjuvant therapy based on the current guidelines and to complete clinical examinations every 3–6 months during the first 2 years and then every 6 months to 1 year thereafter. Disease-specific events were defined as locoregional recurrence, contralateral breast cancer, and distant metastasis. Disease-free survival (DFS) was defined as the time from curative surgery to the first instance of a disease-specific event or the last follow-up. Metastasis-free survival (MFS) was defined as the time from curative surgery to the first instance of distant metastasis or the last follow-up.

Continuous variables were expressed as mean ± standard deviation and compared using the Mann-Whitney U-test. Categorical variables were expressed as number (%) and compared using the chi-squared test. Survival curves were plotted using the Kaplan-Meier method and compared using the log-rank test. All analyses were performed using IBM SPSS software (version 21.0; IBM Corp., Armonk, NY) and differences were considered statistically significant at *P*-values of <.05.

## 3 Results

The 715 eligible patients included 257 patients (35.9%) who had axillary LN metastasis, which was classified as N0 (458 patients, 64.1%), N1 (148 patients, 20.7%), N2 (54 patients, 7.3%), or N3 (55 patients, 7.7%). Table 1 shows the clinicopathological characteristics at the time of surgery according to LN status. Patients with N+ disease had larger tumor sizes and higher histological grades than patients with N0 disease, although there were no inter-group differences in hormone receptor or HER-2 status. Among the 503 patients with estrogen and/or progesterone receptor positivity, only 11 patients (2.2%) did not receive hormone therapy. There was no difference in adjuvant hormone treatment according to LN status.

**Table 1:** Demographic and clinical characteristics of the patients according to initial lymph node status.

	All patients (n = 715)	No lymph node metastasis (n = 458)	Lymph node metastasis (n = 257)	*p*-value
Age, mean ± SD	51.88 ± 11.23	51.92 ± 11.18	51.79 ± 11.33	.88
<50 years, n (%)	335 (46.9%)	208 (45.4%)	127 (49.4%)	.31
≥50 years, n (%)	380 (53.1%)	250 (54.6%)	130 (50.6%)	
Tumor size, mean ± SD	2.06 ± 1.33	1.74 ± 1.10	2.64 ± 1.52	<.001
<2 cm, n (%)	426 (59.6%)	315 (68.8%)	111 (43.2%)	<.001
≥2 cm, n (%)	289 (40.4%)	143 (31.2%)	146 (56.8%)	
Estrogen receptor status				.46
Negative	241 (33.7%)	159 (34.7%)	82 (31.9%)	
Positive	470 (65.7%)	296 (64.6%)	174 (67.7%)	
Unknown	4 (0.6%)	3 (0.7%)	1 (0.4%)	
Progesterone receptor status				.24
Negative	310 (43.3%)	206 (45.0%)	104 (40.5%)	
Positive	401 (56.1%)	249 (54.3%)	152 (59.1%)	
Unknown	4 (0.6%)	3 (0.7%)	1 (0.4%)	
HER-2 status				.93
Negative	521 (72.9%)	335 (73.1%)	186 (72.3%)	
Positive	158 (22.1%)	101 (22.1%)	57 (22.2%)	
Unknown	36 (5.0%)	22 (4.8%)	14 (5.5%)	
Histological grade				.04
1–2	409 (57.2%)	270 (59.0%)	139 (54.1%)	
3	246 (34.4%)	142 (31.0%)	104 (40.5%)	
Unknown	60 (8.4%)	46 (10.0%)	14 (5.4%)	
Initial hormone therapy^∗^				.37
Tamoxifen	316 (62.8%)	205 (64.9%)	111 (59.4%)	
Aromatase inhibitor	176 (35.0%)	103 (32.6%)	73 (39.0%)	
None	11 (2.2%)	8 (2.5%)	3 (1.6%)	
Surgery				<.001
Conservation	427 (59.7%)	303 (66.2%)	124 (48.2%)	
Mastectomy	288 (40.3%)	155 (33.8%)	133 (51.8%)	
Disease-specific events				
No	614 (85.9%)	412 (90.0%)	202 (78.6%)	<.001
Yes	101 (14.1%)	46 (10.0%)	55 (21.4%)	
Locoregional recurrence only	14 (13.9%)	11 (23.9%)	3 (5.5%)	.01
Contralateral breast cancer only	9 (8.9%)	6 (13.0%)	3 (5.5%)	
Distant metastasis only	59 (58.4%)	20 (43.5%)	39 (70.9%)	
Combined events	19 (18.8%)	9 (19.6%)	10 (18.2%)	

SD = standard deviation.

∗ Among the 503 patients who were positive for estrogen or progesterone receptors.

During a mean follow-up period of 69.22 months, 101 patients (14.1%) experienced disease-specific events. Only locoregional recurrence was more prevalent among N0 patients than among N+ patients (2.4% vs. 1.2%), and distant metastasis was more prevalent among N+ patients than among N0 patients (6.4% vs. 19.1%) (Table 1).

**Figure 1:**
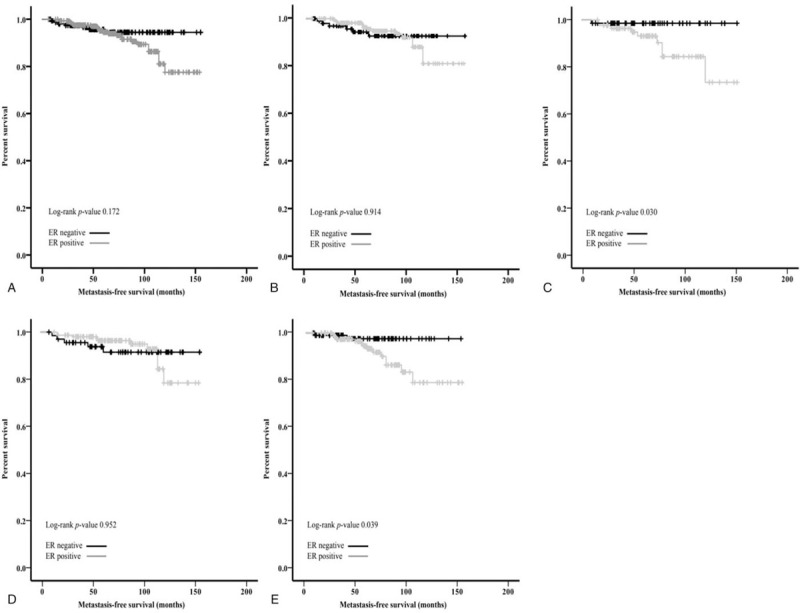
Metastasis-free survival according to estrogen receptor status in patients with lymph node-negative breast cancer. Outcomes are shown (A) among all node-negative patients, among patients with tumor size of <2 cm (B) or ≥2 cm (C), and among patients who were <50 years old (D) or ≥50 years old (E).

Analysis of MFS according to initial LN status revealed 5-year MFS rates of 92.6% among N0 patients and 80.0% among N+ patients, and 10-year MFS rates of 81.8% among N0 patients and 73.5% among N+ patients. Table 2 shows the results of the survival analyses, which revealed that, in the N+ group, poor MFS was associated with large tumor size, ER positivity, and higher histological grade. Among N0 patients, ER positivity was associated with non-significantly shorter mean MFS (ER-positive: 138.90 months vs. ER-negative: 146.99 months, *p* = .17). The 5-year MFS rate was relatively favorable among patients with N0 ER-positive disease (91.7%), although the 10-year MFS rate was only 74.5% and was similar to that of N+ patients (73.5%) (Fig. 1A). Among N+ patients, the 10-year MFS rates were 75.0% for ER-positive patients and 71.5% for ER-negative patients.

**Table 2:** Initial tumor characteristics and metastasis-free survival.

	Lymph node negative group			Lymph node positive group		
	Survival (mean ± SD, months)	Log-rank *P*-value	Univariate HR (95% CI)	Survival (mean ± SD, mo)	Log-rank *P*-value	Univariate HR (95% CI)
Age		.52			.75	
<50 years	143.68 ± 2.83		Ref.	124.10 ± 4.78		Ref.
≥50 years	139.38 ± 3.16		1.26 (0.62–2.57)	111.32 ± 4.59		1.10 (0.624–1.92)
Tumor size		.85			.006	
<2 cm	143.16 ± 2.55		Ref.	133.91 ± 4.42		Ref.
≥2 cm	138.93 ± 3.71		1.08 (0.51–2.29)	113.10 ± 5.25		2.39 (1.27–4.51)
Estrogen receptor status		.17			.01	
Negative	148.28 ± 2.48		Ref.	114.62 ± 6.79		Ref.
Positive	139.25 ± 2.92		1.78 (0.77–4.14)	126.53 ± 4.05		0.50 (0.28–0.88)
Progesterone receptor status		.25			.5	
Negative	146.99 ± 2.52		Ref.	123.73 ± 5.44		Ref.
Positive	138.90 ± 3.24		1.55 (0.73–3.31)	123.38 ± 4.41		0.82 (0.46–1.46)
HER-2 status		.44			.81	
Negative	144.30 ± 2.51		Ref.	122.06 ± 4.22		Ref.
Positive	137.66 ± 4.72		1.38 (0.61–3.16)	124.25 ± 7.16		0.92 (0.47–1.81)
Histological grade		.26			.04	
1–2	141.12 ± 3.17		Ref.	128.39 ± 4.59		Ref.
3	137.24 ± 3.47		1.51 (0.74–3.10)	113.21 ± 3.69		1.80 (1.10–3.22)

CI = confidence interval, HER-2 = human epidermal growth factor receptor 2, SD = standard deviation.

The outcomes among patients with N0 ER-positive disease were evaluated according to patient age at diagnosis and tumor size. Among these patients, poor outcomes were significantly associated with larger tumor size (≥2 cm, *P* = .03) (Fig. 1B and C) and older age (≥50 years, *P* = .03) (Fig. 1D–E).

## 4 Discussion

The present study revealed that ER positivity predicted a poor prognosis among patients with LN-negative breast cancer, and especially among older patients or patients with larger tumors. This information may help physicians predict the course of disease progression and select a treatment and follow-up strategy that balances the benefits, risks, and costs for the patient.

Estrogen plays critical roles in regulating the progression of several cancer types, including breast cancer, and the fate of cancer stem cells.^[8,9]^ In this context, ER-positive cancers are responsive to endocrine therapies and sensitive to CDK4/6 inhibitors,^[10,11]^ which suggests that ER positivity may be associated with a better prognosis. In contrast, ER-negative tumors are more aggressive and tend to metastasize.^[12,13]^ While adjuvant endocrine therapy prolonged the time to recurrence among ER-positive patients,^[14–16]^ patients who received tamoxifen had cumulative recurrence rates that increased from 15% at 5 years to 33% at 15 years and cumulative cancer mortality rates that increased from 8.3% at 5 years to 26% at 15 years.^[14]^

In the present study, most patients with hormone receptor-positive status received hormone therapy, and 97.5% of N0 patients received adjuvant hormone therapy (typically for 5 years). Interestingly, similar 5-year MFS rates were observed for ER-positive and ER-negative patients with N0 disease. Furthermore, patients with N0 ER-positive disease continued to experience distant metastasis at >5 years, which resulted in a prognosis that was similar to that of N+ patients, and this trend was more pronounced among older patients (≥50 years) and patients with large tumors (≥ 2 cm).

A previous study^[17]^ evaluated prognostic factors according to LN, hormone receptor, and HER-2 statuses among patients with early breast cancer who were followed for 20 years. Although that study did not involve a direct comparison, N0 patients with ER-positive/HER-2-positive disease had DFS rates of 80.4% at 5 years and 57.5% at 10 years, which were slightly lower than the rates among patients with ER-negative/HER-2-positive disease (5-year DFS: 85%, 10-year DFS: 58.5%). Moreover, ER-positive/HER-2-positive patients had overall survival (OS) rates of 87% at 5 years and 64% at 10 years, which were lower than the rates among ER-negative/HER-2-positive patients (5-year OS: 95%, 10-year OS: 83%).^[17]^ Another study evaluated patients with N0 ER-positive/HER-2-negative disease according to PR status and Ki-67 index, which revealed poor DFS and a potential benefit from chemotherapy in the low PR/high Ki-67 subgroup.^[18]^

It is possible that prolonged endocrine therapy may improve long-term recurrence and mortality rates, although the IDEAL trial revealed that extended hormone therapy using letrozole (5 years vs. 2.5 years) did not significantly prolong DFS or OS.^[19]^ In addition, the Scottish and NSABP-B14 trials failed to detect significant improvements in DFS or OS after prolonged tamoxifen treatment.^[20,21]^ Nevertheless, the recent ATLAS and aTTom trials clearly demonstrated a better prognosis after extended tamoxifen treatment (10 years vs. 5 years) in large samples of patients.^[22,23]^ Moreover, the ABCSG-6a, MA 17, and NSABP B33 trials indicated that prolonged DFS was observed after 5 years of tamoxifen treatment followed by extended treatment using aromatase inhibitors (AIs).^[24–26]^

A recent meta-analysis^[27]^ revealed that extended endocrine treatment for 10 years could prolong DFS among patients with early breast cancer, especially among ER-positive and postmenopausal patients who received tamoxifen and/or AIs for 5 years followed by AIs for 5 years. That study also revealed that women with N+ disease seemed to experience a greater benefit from extended endocrine therapy (hazard ratio: 0.58, 95% confidence interval: 0.45–0.75).^[27]^ Similarly, other meta-analyses revealed that extended endocrine therapy provided greater benefits among women with N+ disease, larger tumors, and tumors that were positive for ER and progesterone receptor.^[28]^ Nevertheless, these factors may also reflect more serious disease, which suggests that those findings highlight an association between greater risk and greater clinical benefit.

This study had several limitations. First, the retrospective design highlights the need for validation in prospective studies. Second, the study included a relatively small sample of patients with a small number of disease-specific events. Third, the mean follow-up of 69 months was not sufficient for evaluating long-term outcomes. Finally, we were unable to evaluate the different treatment agents and/or treatment periods.

In conclusion, this study revealed that patients with LN-negative ER-positive breast cancer have a risk of metastasis that increases over time, especially among older patients and patients with larger tumors. Therefore, these patient subgroups may require more prolonged follow-up after surgery.

## Author contributions

**Conceptualization:** Eun Jung Jung, Ju-Yeon Kim, Jae-Myung Kim.

**Data curation:** Ju-Yeon Kim, Seung-Jin Kwag, Ji-Ho Park, Soon-Chang Hong.

**Formal analysis:** Ju-Yeon Kim, Jae-Myung Kim, Han Shin Lee.

**Methodology:** Han Shin Lee, Seung-Jin Kwag, Young-Joon Lee, Soon-Chang Hong.

**Project administration:** Young-Tae Ju.

**Supervision:** Ji-Ho Park, Taejin Park, Sang-Ho Jeong, Chi-Young Jeong, Young-Tae Ju, Young-Joon Lee.

**Validation:** Taejin Park, Sang-Ho Jeong, Chi-Young Jeong.

**Writing – original draft:** Ju-Yeon Kim.

**Writing – review & editing:** Eun Jung Jung, Ju-Yeon Kim.
